# A Bioguided Approach for the Screening of Antibacterial Compounds Isolated From the Hydroalcoholic Extract of the Native Brazilian Bee’s Propolis Using Mollicutes as a Model

**DOI:** 10.3389/fmicb.2020.00558

**Published:** 2020-04-07

**Authors:** Sabrina Hochheim, Pamela Pacassa Borges, Ariela Maína Boeder, Dilamara Riva Scharf, Edésio Luiz Simionatto, Celina Noriko Yamanaka, Michele D. Alberton, Alessandro Guedes, Caio Mauricio Mendes de Cordova

**Affiliations:** ^1^Graduate Chemistry Program, Universidade de Blumenau – FURB, Blumenau, Brazil; ^2^Biomedicine School, University of Blumenau – FURB, Blumenau, Brazil; ^3^Pharmacy School, University of Blumenau – FURB, Blumenau, Brazil; ^4^Department of Chemistry, University de Blumenau – FURB, Blumenau, Brazil; ^5^Department of Pharmaceutical Sciences, University of Blumenau – FURB, Blumenau, Brazil

**Keywords:** propolis, Brazilian native bees, bioguided fractioning, mollicutes, mycoplasmas, antimicrobials

## Abstract

Nature is a vast source of medicinal substances, including propolis, which has been extensively investigated. Propolis is a resinous substance produced by bees from the exudates of plants that they collect and modify in their jaws; it is a rich and complex matrix with secondary metabolites of diverse botanical origins. The objective of this study was to apply an *in vitro* bioguided approach using as a model system the mollicutes with a sample of propolis from the Brazilian native bee *Melipona quadrifasciata* (mandaçaia) in order to identify potential new molecules with antimicrobial activity. A crude hydroalcoholic extract was obtained and submitted to liquid-liquid partitioning with solvents of different polarities, generating four different fractions: aqueous, dichloromethane, butanol, and ethyl acetate fractions. The antimollicute activity assays served as a basis for the bioguided fractionation. The dichloromethane fraction was the most promising, exhibiting a minimal inhibitory concentration (MIC) of 125 μg/mL against *Mycoplasma pneumoniae*. After purification by column liquid chromatography, a subfraction presenting MIC of 15.6 μg/mL against *Mycoplasma genitalium* was highlighted. The fractions were also tested against *Escherichia coli*, *Staphylococcus aureus*, and *Pseudomonas aeruginosa*. Using gas chromatography coupled to a mass spectrometer (GC-MS), several volatile compounds were identified in the non-polar fractions of this propolis. However, the more purified molecules had no better antimollicute activity than their original subfraction. Apparently, the synergism among its compounds is largely responsible for the antibacterial activity of the propolis of this native Brazilian bee.

## Introduction

Bees are able to explore unique sites and found the best molecules for their benefit, producing propolis to protect their hive (Toreti et al., 2013; Bankova et al., 2014). Being a mixture of secondary metabolites of the flora that surrounds the hive, propolis may thus be an important matrix that can be exploited for many therapeutic purposes (Pobiega et al., 2018).

Due to its chemical complexity, each type of propolis must be studied individually, and one of the greatest challenges concerning the medicinal application of propolis is the standardization of the minimum quality characteristics of the extracts obtained from it so that its therapeutic applications can be better utilized (Sforcin and Bankova, 2011). In this aspect, bioguided tests with propolis extracts can provide researchers with relevant information to help them decide whether to intensify the purification of fractions. The isolation of new compounds and the determination of their chemical structures is facilitated because only fractions of greater biological relevance are further investigated (Bankova, 2005).

Bioguided tests are those that deal primarily with complex samples, in which some prior fractionation is necessary to reduce their complexity. Once a biologically active fraction or even an isolated compound with a demonstrated biological interaction has been found in a living organism of interest, the chemical analysis to determine the structure and physical-chemical properties can be carried out (Weller, 2012).

This type of assay can be used in the search for new antimicrobial agents since the need to combat bacterial resistance is obvious and urgent. In this quest, the mollicutes constitute a cellular and molecular biological model of interest. Due to the shortening of their genome throughout evolution, the vast majority of mollicutes have the smallest genomes capable of self-replication and retain only sequences of metabolic pathways that are essential for their survival (Fisunov et al., 2011). Thus, mollicutes can be used as models for the investigation of new molecules with antimicrobial potential, and it is easier to establish their mechanism of action in organisms with small genomes than in organisms with genomes that encode several thousands of genetic products (Chernov et al., 2018). Considering that mollicutes are the microorganisms that have the lowest number of genes necessary for self-replicating life, thus mostly essential genes, we hypothesize that new compounds that present significant antimicrobial activity against mollicutes have the potential to be active against other types of bacteria, even the multiresistant ones. Therefore, the objective of this study was to apply a bioguided approach, using the mollicutes as a model system, to test a sample of propolis from the native Brazilian bee Melipona quadrifasciata (mandaçaia), in order to identify possible new molecules with antimicrobial activity.

## Materials and Methods

### Propolis Sample

The sample of propolis, previously classified as yellow-green propolis (Hochheim et al., 2019), was obtained from the hives of the native species *Melipona quadrifasciata* in southern Brazil (26°54′21.3″ S, 49°04′49.1″ W).

### Propolis Extracts

The sample was pulverized and macerated in 70% ethanol, transferred to a vial and conditioned in a dark room for 7 days, and then filtered under *vacuum* and brought to complete drying on a rotary evaporator under reduced pressure. A portion of 50 g of the so-treated sample was solubilized in water and submitted to liquid-liquid partitioning employing solvents of different polarities (dichloromethane, butanol, and ethyl acetate), generating 4 fractions: aqueous (FAq), dichloromethane (FDi), butanol (FBu), and ethyl acetate (FAc).

### Bioguided Fractioning of the *M. quadrifasciata* Propolis

The antimicrobial activity against mollicute strains (described below) was used as a guide for the bioguided fractionation. In this way, FDi was fractionated through a silica gel chromatographic column. For this, solvent grade P.A. silica gel with a particle size of 70–200 mesh (column 1) or 230–400 mesh (other columns) (Vetec^®^, Rio de Janeiro, Brazil) was used, and for thin-layer chromatography (TLC), aluminum plates coated with silica gel 60 F254 (Merck^®^, Darmstadt, Germany) were used. An aliquot of 15 g of the FDi was subjected to silica gel column chromatography (17 × 6.5 cm) (column 1) employing eluents in increasing degree of polarity. Fifty subfractions of 125 mL each were collected, and the solvent was eliminated in a rotary evaporator. The subfractions were analyzed by TLC and assembled according to their chromatographic similarity, resulting in 20 final subfractions. Of these, the ones with the most distinct TLC profiles (FM6, FM9, FM14, FM24, FM34, and FM45) were selected for the antibacterial assay against mollicute strains, and those with a higher degree of purity (fewer spots) on the TLC (FM14 and FM24) were analyzed by GC-MS. An aliquot of 1.34 g of the subfraction FM9 was chromatographed on a silica gel flash column (17 × 3 cm) (column 2). Ten subfractions of 10 mL each were collected and analyzed by TLC. The subfractions were assembled according to their similarity, which resulted in 6 final subfractions (FM9-01, FM9-16, FM9-28, FM9-35, FM9-41, and FM9-50). Purposing to obtain a higher degree of purity of the subfractions, a 0.184 g aliquot of the FM9-16 subfraction was subjected to silica gel (15 × 2 cm) flash column chromatography (column 3). Eight subfractions of 10 mL each were analyzed by TLC and assembled according to their similarity, resulting in 10 subfractions, of which 2 were considered to be of higher purity (SH-9 and SH-19) and submitted to GC-MS analysis (see below). The FM45 subfraction was also considered to have potential antimicrobial activity against mollicutes, and therefore it was submitted to a new purification. Gradual mixtures of chloroform and methanol were used as eluents, starting from 100% chloroform to 100% methanol. Fifty-five subfractions were collected, analyzed by TLC, and assembled according to their degree of similarity, resulting in 10 final subfractions. From that, the ones with the highest degree of purity (FM45-44 and FM45-56) were selected to be tested again for their antimicrobial activity.

### Gas Chromatography Coupled to Mass Spectrometry

The qualitative analyses were performed by GC-MS, QP2010 Plus Shimadzu^®^, using columns RTx-5MS 30 m × 0.25 mm × 0.25 μm. For analysis of the subfractions FM14 and FM24, a heating ramp of 60°C was used for 5 min, with a rise of 3°C/min to 300°C for 13 min, and for the SH-9 and SH-19 subfractions, a heating ramp of 100°C was used for 10 min, with a rise of 12°C/min to 300°C for 24 min. The injector temperature was 250°C with a split of 1:20, a source of ions at 250°C in the MS with an interface at 280°C, and a flow of helium gas at 1 mL/min. The identification of the propolis components and its subfractions, when possible, was made by comparing their mass spectra with the NIST^®^ 2014 database.

### Antimicrobial Activity Against Strains of Bacteria Without Cell Wall (Mollicutes)

The microorganisms used in this work were the strains *Mycoplasma hominis* ATCC 23114, *M. capricolum* ATCC 27343, *M. genitalium* ATCC 33530, *M. pneumoniae* FH (ATCC 15531), and *M. mycoïdes* subsp. *capri* PG3 (NCTC 10137). Minimum inhibitory concentration (MIC) assays were performed by the broth microdilution method on 96-well plates as indicated by the Clinical and Laboratory Standards Institute (Clinical Laboratory Standards Institute [CLSI], 2011) with minor modifications. The crude extract and fractions were diluted to 40 mg/mL, and the purified subfractions were diluted to 4 mg/mL in dimethylsulfoxide (DMSO 100%). As a negative control, a serial dilution of the solvent was used (DMSO 100%), without the presence of the extracts; as a growth control, a serial dilution of the culture of the microorganism was used, without the addition of solvent or propolis extract; as a positive control, the antibiotic azithromycin (DME^®^, Araçatuba, Brazil) was used; and as a control of sterility, a cavity was reserved for each sample and filled with culture medium. Finally, two to three drops of liquid sterile mineral oil were added in all the wells in order to isolate each cavity from the external environment. Plates were incubated at 37°C for the time required for each strain (1–30 days), and growth was observed from the color change of the culture medium, due to the presence of the phenol red pH indicator. Three replicates were performed for each test, on different days and in a laminar flow hood.

### Antimicrobial Activity Against Strains of Gram-Positive and Gram-Negative Bacteria

The strains *Staphylococcus aureus* ATCC 25923, *Escherichia coli* ATCC 25922, and *Pseudomonas aeruginosa* ATCC 27853 were used. MIC determination was performed by the broth microdilution technique in 96-well microplates, as recommended by the Clinical Laboratory Standards Institute [CLSI] (2012). Samples diluted to 2 mg/mL in dimethyl sulfoxide (DMSO 100%) were placed in the first wells and then transferred to the adjacent wells, which already contained Muller–Hinton (MH) broth, to obtain serial two-fold dilutions, with sample concentrations ranging from 1,000 to 7.81 μg/mL. The bacterial inoculum was prepared on the McFarland 0.5 scale (5 × 105 CFU/mL), and 5 μL was added to each well. Some of the wells of each microplate were reserved for negative controls (MH + H_2_O/DMSO + bacterial inoculum) and sterility of the culture medium (MH only). As a positive control, the antibiotic gentamicin was used in concentrations ranging from 40 to 0.31 μg/mL. Three replicates were performed for each test, on different days and in a laminar flow hood. The microplates were incubated aerobically at 37°C ± 1°C for 24 h. After incubation, the bacterial growth was verified by adding 10 μl of a methanolic solution of 2,3,5-triphenyltetrazolium chloride (5 mg/mL) to each well. After 2 h, the formation of a reddish bacterial “button” at the bottom of each well indicated the viability of the bacterium. MIC was determined as the last concentration capable of inhibiting bacterial growth.

### Statistical Analysis

The significance of the antimicrobial test results were subjected to analysis of variance (ANOVA), with a confidence level of 95%, using the ezAnova software (Chris Rorden© 2007).

## Results

For biomonitoring purposes, we used the cut-off point proposed by Machado et al. (2005), in which extracts and fractions with MICs below 10 μg/mL are considered excellent, MICs between 10 and 100 μg/mL are considered good, MICs from 100 to 500 μg/mL are considered moderate, MICs of 500–1,000 μg/mL are considered weak, and MICs above 1,000 μg/mL are considered inactive. The results of the activity of the extracts studied against the mollicute strains are shown in Table 1.

**TABLE 1:** MIC of the crude extract of propolis and fractions expressed in μg/mL against different bacteria without cell wall (mollicutes).

**Fraction/strain**	***M. genitalium***	***M. capricolum***	***M. pneumoniae***	***M. hominis***	***M. mycoïdes***
PC	2^a,a^	2^a,a^	2^a,a^	2^a,a^	2^a,a^
EBH	250^b,a^	500^b,a^	250^b,a^	250^b,a^	500^b,a^
FAq	>1,000^c,a^	>1,000^c,a^	>1,000^c,a^	>1,000^c,a^	>1,000^c,a^
FDi	250^b,a^	250^b,a^	125^b,a^	250^b,a^	500^b,a^
FAc	500^b,a^	500^b,a^	500^b,a^	500^b,a^	1,000^c,a^
FBu	>1,000^c,a^	>1,000^c,a^	>1,000^c,a^	1,000^c,a^	1,000^c,a^

*PC = positive control (azithromycin). EBH, crude hydroalcoholic extract; FAq, aqueous fraction; FDi, dichloromethane fraction; FAc, ethyl acetate fraction; FBu, butanol fraction. Data with equal uppercase letters in the same column (before the comma) or in the same line (after the comma) mean no statistical difference among each other (*p* > 0.05); in a confidence interval of 95%.*

EBH and FDi presented better results, and the FDi was chosen to be purified (MIC of 125 μg/mL against *M. pneumoniae*).

Concerning the bacteria with the cell wall, it can be observed (Table 2) that *E. coli* was sensitive to FDi at a concentration of 125 μg/mL, and the EBH presented a MIC of 250 μg/mL. Against *P. aeruginosa* the EBH and FDi presented a MIC of 125 μg/mL. *S. aureus* showed the highest susceptibility to EBH and its fractions. EBH and FDi reached a MIC of 125 μg/mL, and FBu presented a MIC of 15.62 μg/mL against *S. aureus*.

**TABLE 2:** MIC from the crude extract of propolis and fractions expressed in μg/mL against Gram-positive and Gram-negative bacteria.

**Fraction**	***E. coli***	***S. aureus***	***P. aeruginosa***
EBH	250^a,a^	125^a,a^	125^a,a^
FAq	>1,000^b,a^	>1,000^b,a^	>1,000^b,a^
FDi	125^a,a^	125^a,a^	125^a,a^
FAc	>1,000^b,a^	1,000^b,a^	>1,000^b,a^
FBu	>1,000^b,a^	15.62^c,b^	>1,000^b,a^
PC	20^c,a^	20^c,a^	20^c,a^

*PC = positive control (gentamicin). EBH, crude hydroalcoholic extract; FAq, aqueous fraction; FDi, dichloromethane fraction; FAc, ethyl acetate fraction; FBu, butanol fraction. Data with equal uppercase letters in the same column (before the comma) or in the same line (after the comma) mean no statistical difference among each other (*p* > 0.05); in a confidence interval of 95%.*

**FIGURE 1:**
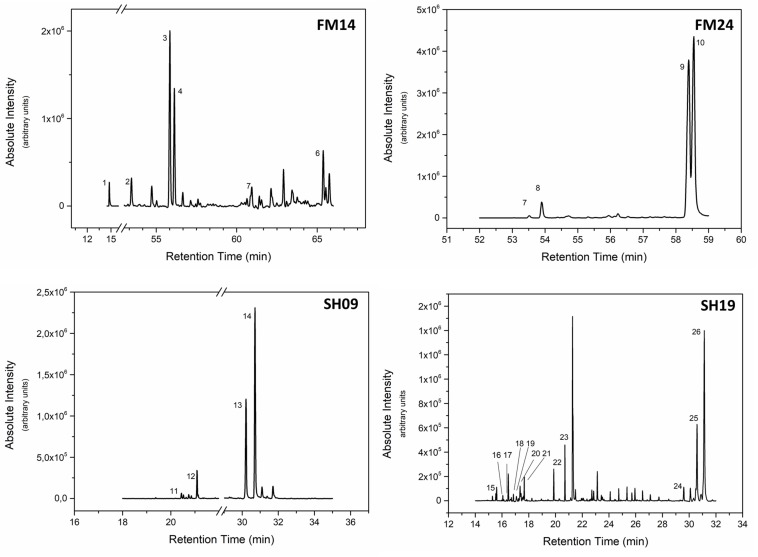
GC-MS spectra of the subfractions FM14, FM14, SH09, and SH19 obtained from *M. quadrifasciata* propolis extracts.

To correlate the antimicrobial activities and the chemical composition of propolis, purified subfractions (FM14, FM24, SH-9, and SH-19) were submitted to GC-MS (Figure 1). In these fractions, it was possible to identify the presence of one monoterpene alcohol, sesquiterpenes, sesquiterpene ketones, diterpenes, triterpenes, and saturated and unsaturated fatty acids. The detailed description of the molecules identified is given in Table 3.

**TABLE 3:** Volatile components identified by GC-MS in the purified subfractions of propolis.

**Analyzed subfraction/fraction of origin**					
**FDi**					
	**Peak**	**Molecule**	**RT**	**M/Z**	**Area (%)**
FM14	1	cis-Verbenol	14.78	94, 109, 59	4.44
	2	β-Elemene	52.66	93, 81, 68	1.77
	3	Thunbergol	55.84	81, 95, 107	47.15
	4	Lanosterol acetate	56.13	95, 55, 81	31.5
	5	Shonanol	63.09	285, 243, 300	0.85
	6	Sugiol	65.39	285, 300, 217	14.28
FM24	7	β-Elemene	53.51	93, 81, 68	0.62
	8	Viridiflorol	56.23	69, 122, 109	0.67
	9	Agathadiol	58.39	81, 95, 153	48.95
	10	Cycloartanyl acetate	58.56	95, 81, 55	49.75

**SH-9**					
FM09 SH-9	11	β-Elemene	20.44	81, 93, 121	0.97
	12	Epimanool	21.08	137, 81, 95	4.94
	13	α-Amyrin acetate	30.22	218, 203, 189	32.63
	14	α-Amyrin	30.70	218,189, 203	61.46
FM09 SH-19	15	Cubebol	15.28	161, 105, 119	0.72
	16	Elemol	16.06	59, 93,161	0.73
	17	Spathulenol	16.46	119, 205, 91	0.1
	18	Guaiol	16.69	161, 107, 204	0.37
	19	Rosifoliol	16.85	149, 59, 108	0.91
	20	β-Eudesmol	17.34	59, 149, 108	1.32
	21	Aristolone	17.66	147, 105, 91	2.5
	22	Palmitic acid methyl ester	19.86	74, 87, 143	4.6
	23	Linoleic acid methyl ester	21.28	67, 81, 95	19.86
	24	Lanosterol	29.58	411, 69, 109	2.56
	25	β-Amyrin	30.58	218,189, 203	15.84
	26	α-Amyrin	31.13	218,189, 203	50.40

The FM45 subfraction, also obtained from this first fractionation of propolis, was previously analyzed by HPLC-ESI-MS/MS. This subfraction was demonstrated to be a mixture of aromadendrin, *p*-coumaric acid, naringenin, catechin, epicatechin, and pinocembrin (Hochheim et al., 2019). There was an interest in identifying the components of this subfraction once it had reached a MIC of 15.62 μg/mL against *M. genitalium*. After the purification of this subfraction, two new subfractions were obtained: SH44 and SH56. These were again tested against *M. genitalium*, starting from a concentration of 4 mg/mL with a MIC of 100 μg/mL for the SH44 subfraction, while SH56 was not effective at this concentration (Table 4).

**TABLE 4:** Inhibitory Minimal Concentration (MIC) of the subfractions of propolis expressed in μg/mL against bacteria without cell walls (mollicutes).

**Subfraction**	***M. genitalium***	***M. capricolum***	***M. pneumoniae***	***M. hominis***
FM06	250^a,a^	500^a,b^	250^a,a^	500^a,b^
FM09	250^a,a^	500^a,b^	125^a,a^	500^a,b^
FM14	500^b,b^	500^a,b^	250^b,a^	250^b,a^
FM24	500^b,b^	500^a,b^	250^b,a^	250^b,a^
FM34	500^b,b^	500^a,b^	250^b,a^	250^b,a^
FM45	15.62^c,c^	250^b,b^	125^d,a^	125^d,a^
SH-9	50^c^	NT	NT	NT
SH-19	>100	NT	NT	NT
SH-44	100	NT	NT	NT
SH-56	>100	NT	NT	NT
PC	2	2	2	2

*NT = not tested; PC = positive control (azithromycin). Data with equal uppercase letters in the same column (before the comma) or in the same line (after the comma) mean no statistical difference among each other (*p* > 0.05); in a confidence interval of 95%.*

## Discussion

Mollicutes are excellent models for studying the mechanisms of action of new drugs since they conserve mainly essential metabolic pathways. Thus, it is more likely that new antimicrobials acting on mollicutes may also act on other bacteria, and it is easier to establish the mechanisms of action of new drugs in an organism with a tiny genome (Chernov et al., 2018). In this study, we have demonstrated significant antimollicute activity of several subfractions purified from propolis of the Brazilian native bee *M. quadrifasciata*.

A singular sample of propolis may contain more than 420 compounds, such as phenolic acids and their esters, flavonoids, chalcones, and dihydrochalcones, terpenoids, acyclic hydrocarbons and esters of higher alcohols, alcohols, aldehydes, amino acids, aromatic hydrocarbons, fatty acids, ketones, sterols, sugars (Opsenica et al., 2016). Due to its complexity, a prior fractionation, using solvents of different polarities, assists in the identification of the components of each sample, facilitating the use of different characterization techniques. In this study, the biomonitoring fractionation of the non-polar propolis compounds extracted with dichloromethane was chosen since its MIC results against *M. pneumoniae* proved to be more promising.

The volatile components of propolis vary greatly in each sample, and their study contributes significantly to the understanding of its pharmacological properties. It is noteworthy that in several samples of propolis from France, Hungary, Bulgaria, and Italy, the sesquiterpene β-eudesmol was demonstrated to be the major component (Bankova et al., 2014). Fatty acids and long-chain alkanes have already been reported in several samples of propolis (Czyżewska et al., 2015). Mono-, sesqui-, and diterpenes labdanes are often found in green propolis, contributing to its resinous features, to its pleasant odor, and probably to its antimicrobial properties (Salatino et al., 2005). The presence of the terpene components in the studied propolis gives an idea of the complexity of its composition and instigates deeper research on the contribution of each component in the antimicrobial activity.

From the results obtained with the subfraction SH-9, we can see that in comparison with its original subfraction (FM 09), it presented a lower MIC, demonstrating that the compounds present in this particular subfraction, when more purified, can have a more intense level of antimicrobial activity. The antimicrobial activity of the SH-9 fraction may be related to the diterpene labdane manool, which has already been reported in the literature as having very low MICs against strains of oral pathogens (Moreira et al., 2013). Besides, our research group has been testing isolated forms of α- and β-amirin, from other sources, however, no success in antibacterial activity was obtained.

Concerning the activity of SH-44 and SH-56 compounds, no improvement in activity can be observed when compared with its original subfraction (FM45). This can be explained by an eventual synergistic activity among the propolis compounds, and many of them may lose their biological activities when separated (Bankova et al., 2014).

Antibacterial tests are among the most common tests conducted with natural products in the search for new antibiotics (El-Guendouz et al., 2018). In this way, the antimicrobial activity of the propolis of *Apis mellifera* is already well established, and the results against bacteria with a cell wall have already been reported by other authors (Velikova et al., 2000; Silici and Kutluca, 2005; Trusheva et al., 2010). An interesting fact is that several studies have observed a low activity of propolis extracts against Gram-negative bacteria (Popova et al., 2011; Netikova et al., 2013; Przybylek and Karpinski, 2019). Many samples of honeybee propolis sent for bacterial analysis have been shown to have high levels of *E. coli* (Nogueira-Neto, 1997), which could indicate that these bacteria do not harm the colonies, thus accounting for the low activity of propolis against Gram-negative strains.

In contrast, *S. aureus* was more sensitive to EBH and its fractions. Optimal results were obtained with FBu, which reached a MIC of 15.62 μg/mL. This is an important fraction for further investigation. The mollicute bioguided fractionation strategy was not able to provide a promising novel antibiotic from the propolis type used in this study, but the compounds present in the butanol fraction may be quite promising against *S. aureus*. These trials are still ongoing.

The activity of propolis against strains of Gram-positive bacteria has already been proven by several studies, and some authors attribute the antibacterial activity in Brazilian propolis to prenylated *p*-coumaric acid compounds and diterpenes of the labdane type (Bankova, 2005; Monteiro et al., 2014). Others argue that the antimicrobial activity is more related to the flavonoid content or to the synergism between flavonoids, hydroxy acids, and terpene compounds (Marcucci, 1995). This synergistic effect seems to be the most faithful way to describe the biological activities of propolis since it has already been proven that a single isolated component, may have no better effects than its entire extracts or fractions (Pereira et al., 2011).

In this study, it was possible to observe this synergism with the analysis of the subfraction FM45. After a first purification round, this subfraction had a MIC of 15.62 μg/mL (greater than that of FDi) due to a higher concentration of the active components. However, after subsequent purifications, the compounds SH-9 and SH-19 showed no improvement in antibacterial activities.

## Data Availability Statement

The datasets generated for this study are available on request to the corresponding author.

## Author Contributions

SH conducted the majority of the experimental procedures, analyzed and discussed the data. PP and AB contributed in the propolis purification procedures. PP contributed in the antimollicute assays. AB and CY contributed in the cell-walled antibacterial assays. DS and ES contributed in the spectrometric analysis. MA and AG contributed in the propolis purification procedures and characterization. CC designed the study, coordinated its conduction and reviewed the data analysis. All authors contributed to the manuscript elaboration and revised the final version.

## Conflict of Interest

The authors declare that the research was conducted in the absence of any commercial or financial relationships that could be construed as a potential conflict of interest.
